# Forest bathing and hiking benefits for mental health during the COVID-19 pandemic in Mediterranean regions

**DOI:** 10.1007/s10342-023-01531-6

**Published:** 2023-02-03

**Authors:** Anna Muro, Corel Mateo, Eva Parrado, Montse Subirana-Malaret, Montserrat Moya, Adrià Garriga, Josep Canals, Andrés Chamarro, Antoni Sanz

**Affiliations:** 1 Departament of Basic, Developmental and Health Psychology, Autonomous University of Barcelona, Office B5-111, Campus UAB, Bellaterra, 08193 Barcelona, Spain; 2 Serra Húnter Programme, Generalitat de Catalunya, Barcelona, Spain; 3 Sport Research Institute, Universitat Autònoma de Barcelona, Barcelona, Spain; 4 Faculty of Psychology, University of Barcelona, Barcelona, Spain; 5 Cooperativa Sèlvans, La Garrotxa, Girona, Spain; 6 School of Nature La Muntada, Sant Llorenç Savall, Barcelona, Spain; 7 Natural Park of Sant Llorenç, Catalan Net of Natural Parks, Barcelona Provincial Council, Barcelona, Spain

**Keywords:** Forest bathing, COVID-19, Mediterranean forests, Well-being, Mental health

## Abstract

Forest bathing (FB) has evidenced positive effects on individuals’ mental health and well-being, but its benefits have mainly been studied in Asian biomes. The present study aimed to evaluate whether its benefits are also generalisable to other forests and biomes of the world, such as the Mediterranean. Eighty-six healthy adults of the general population were assessed before and after a FB near Barcelona (Spain) during the COVID-19 pandemic. A control-hiking group of participants was also analysed to contrast the FB effects on anxiety, affect, mood states and mindfulness. Results show that the guided practice of FB in Mediterranean-Catalan forests increases mindfulness states and positive affect and reduces anxiety and negative affect, with effect sizes being large to very large. Hiking also induced significant changes in all variables tested, but FB showed higher effect sizes. An exploratory analysis also revealed a different profile of the FB participants compared to the hiking practitioners, being highly educated women living in urban areas and with lower basal levels of psychological well-being. Accordingly, it is concluded that both Mediterranean FB and hiking (to a lesser degree) might be cost-effective strategies to promote and restore psychological well-being after the COVID-19 pandemic and to promote sustainable tourism in Mediterranean biomes of the European forested and protected areas.

## Introduction

Shinrin-Yoku (forest bathing; FB) is a traditional Japanese nature practice which consists in walking by the forest in silence and by paying attention to the one’s senses in connection with nature (Kotera et al. 2020; Miyazaki 2018). Its practice has been spread around the world during the last decade, and it is considered both a recreational and a therapeutic activity, guided and structured, aimed at improving health and well-being (Farkic et al. 2021; Hansen et al. 2017; Wen et al. 2019). It activates human senses, allowing multisensory, affective and kinaesthetic experiences that induce relaxation and vitality. During the last decade, numerous studies performed in Asian biomes have consistently reported the restorative effects of FB for mental health (Hansen et al. 2017; Timko-Olson et al. 2020), showing how walking by a forest in silence reduces stress, depression and anxiety levels. FB is a nature activity mainly practiced in Japan, a country that counts with around 60 forest medicine centres that coordinate with the major health and primary care units of major metropolises (Li 2018; Park et al. 2010). FB provides an added value to forested areas and channel the demands of contact with nature by a growing urban population with intense levels of stress, competitiveness, screen or new technologies addiction and related problems such as sedentarism, obesity and cardiovascular problems (Morita et al. 2007; Oh et al. 2017; Wen et al. 2019). Accordingly, FB has been also regarded as a sustainable touristic activity that enable visitors discover forest landscapes and that promotes pro-environmental attitudes and well-being (Farkic et al. 2021). Empirical evidence suggests that exposition to natural environments improves per se well-being, reduces stress and depressive mood (Antonelli et al. 2019; Park et al. 2010; White et al. 2021). Coherently, walking by the forest in silence adds significant improvements for other health outcomes given the revitalising effect of nature exposure with low physical activity and silence (Chen et al. 2018; Hansen et al. 2017; Kobayashi et al. 2018; Lyu et al. 2018; Li 2018). Thus, FB has also been associated to overall improvements in immune and cardiovascular functioning and to increases in positive emotions and cognitive functions such as attention, as well as to reductions in pain perception (Hansen et al. 2017; Kotera et al. 2020; Li 2018; Wen et al. 2019; Yau and Loke (2020). Some authors suggest that the inhalation of terpenes, as main volatile oils contained in forest aerosols, is the mechanism that mediates the relationship between FB and health improvements (Cho et al. 2017). It is also argued that the specific forest’s biome is also a factor that might mediate these restorative and healing effects for humans’ health (Bach et al. 2020a, b).

Given the mounting evidence of FB’s health benefits, it is considered an evidence-based activity in forest medicine and included as a key element in nature-outdoor therapies (Biedenweg et al. 2017; Bratman et al. 2019). However, although empirical studies in Asian biomes are plentiful, a recent review indicates that more evidence is needed to study the differential impact of FB in other biomes of the world (Kotera et al. 2020), such as the Mediterranean biome. FB practice has grown significantly during recent years in this area, but just a few pilot-empirical studies have been carried out to date exploring its specific effects for health (Hansen et al. 2017; Bach et al. 2021; Muro et al. 2022; Rodríguez 2018; Wen et al. 2019). Unlike Asian mesophytic or deciduous forests, Mediterranean forests are part of a temperate terrestrial biome, characterised by hot, dry summers and rainy winters (Bach et al. 2021; Mucina 2019). Mediterranean forests can be mainly found in big five ecoregions of the world: the Mediterranean Basin, California, central Chile, southwestern Australia and the Western Cape of South Africa. The Mediterranean biome is also called evergreen sclerophyll (scleros = hard, sharp = leaf), as it is mostly made up of hard-leaved perennials (Woodward et al. 2004).

Therefore, the present study was designed to assess the psychological benefits of FB and other hiking activities in Mediterranean forests, more specifically in the protected areas of the Catalan region, at the north-east of the Iberian peninsula. The study also aimed to facilitate the implementation of FB in Catalan forests during the pandemic among urban populations, whose mental health has been significantly hit by the COVID-19 pandemic (Sanabria-Mazo et al., 2021; WHO 2020); finally, it also aimed to replicate a pilot study published during the COVID-19 pandemic exploring the benefits of FB in a Mediterranean-Catalan forest. It reported large increases in positive affect, vigour, friendship and mindfulness and large decreases in negative affect, anxiety, anger, fatigue, tension and depressive mood in sample of 17 healthy adults (Muro et al. 2022). Despite the very large effect sizes reported in this pilot study, methodological issues related with the small sample size, the lack of control group and the lack of forests’ diversity, made it hard to generalise results. Accordingly, the present study increased the sample size, the type of forests and included two control groups of hiking activities to contrast the results of the FB-experimental condition. Hiking, as a nature activity that consists in walking around green landscapes, has also numerous benefits for health, but it is rather considered a touristic- recreational and physical activity (Mitten et al. 2018). Therefore, in an exploratory way, we aimed to study whether there was any self-selection bias between participants who decided to carry out a guided practice of forest bathing vs. a guided practice of hiking, since the first is perceived as a more therapeutic activity while the second is considered a recreational-sports activity. Likewise, given the fact that the study included two different sub-biomes of the Mediterranean-Catalan biome, the differential effect obtained with the practice in both was also explored.

The hypotheses were the following:

### H_1_

FB would significantly increase participants’ positive affect, vigour, friendship and mindfulness and would decrease negative affect, anxiety, anger, fatigue, tension and depressive mood.

### H_2_

There would be also changes in the control-hiking groups in the indicators measured, but changes would be expected to have a higher impact in the FB group.

### H_3_

There would be no differences among the forest bathing effects performed in the two different Mediterranean sub-biomes.

## Materials and methods

### Design and procedure

A pre-post with control group design was used to evaluate the psychological benefits of FB compared with guided hiking in Catalan forests. Most of the Catalan territory lies on the north-east of the Iberian Peninsula, at the south of the Pyrenees Mountain range. The capital and largest city is Barcelona, the second most populated city in Spain and the fifth most populated urban area in the European Union (Demographia 2021).

The intervention followed the procedure employed in a pilot study carried out in May 2020 (Muro et al. 2022), but the current study included some variations, including two experimental conditions distributed in different locations:

- *Experimental group*. It included participants that took a FB with specialised nature guides, in two different main different allocations: (1) Vallès Occidental, natural park of Sant Llorenç and (2) la Garrotxa area, volcanic zone Natural Park. Both spaces are located at a linear distance of 70 km, in the Mediterranean biome of the Mediterranean basin, included in the north-eastern Spain and Southern France Mediterranean forests (Olson and Dinerstein 1998). The groups were conducted by different guides and sub-biomes but both walked by paths near old trees, big stones and little rivers that enriched the participants’ sensory stimulation.
- The *first group* (Sant Llorenç) was recruited in collaboration with the Diputació de Barcelona, a public administration that promotes the progress and well-being of the citizens and local governments of its territorial area, and the Network of Natural Parks of Catalonia (https://parcs.diba.cat), announcing the activity for the general population during the first waves of the pandemic: one for October 2020 (https://parcs.diba.cat/web/agenda/-/sant-lloren%C3%A7-obac-banya-t-al-bosc-experi%C3%A8ncia-immersiva-a-la-m%C3%A0gia-de-les-arenes) and the other for May 2021 (https://parcs.diba.cat/web/agenda/-/sant-lloren%C3%A7-obac-banya-t-al-bosc-experi%C3%A8ncia-immersiva-a-la-m%C3%A0gia-de-les-arenes-1). The activity was framed within the *Ginesta project* of environmental education, included in projects of the European Charter for Sustainable Tourism for 2021, which is a set of good environmental practices for the management of tourism in protected natural areas, promoted by the Europarc Federation (Canals 2014; Europarc Federation 2010).
- The forested area chosen for the FB was in Les Arenes de Sant Llorenç del Munt (geographical coordinates: N 41° 38.799 N, E 2° 3.457): it is an area of a mountain Mediterranean forest in Sant Llorenç del Munt i Serra de l’Obac Natural Park (Catalonia, north-east of the Iberian Peninsula), a massif included in the central pre-coastal Mediterranean climate, close to urban centres. The base of the massif is occupied by white pine groves (Pinus halepensis), very resistant to water scarcity and up to 600 masl, often being replaced in the darkest and/or tallest areas by Pinus sylvestris and Pinus nigra. These pine forests, for the most part, are the result of the human transformation of the primitive forest and in many areas appear mixed with holm oaks (Quercus ilex) and Mediterranean shrubs such as heather (Erica) and strawberry (Arbutus unedo). The holm oak groves are the characteristic and most widespread vegetation of the natural park, which above 800 masl is enriched with species typical of wetlands such as whitebeam (Sorbus), boxwood (Buxus) and oak (Quercus humilis) (Lorenzo and Fernández; 2009). The walk included 2 stops (one at the beginning and one at the middle of the walk) to practice short mindfulness-meditations of 10 minutes each and train deep breathing and mindful attention. Participants were asked to be aware and open their senses to facilitate nature connectedness (by listening the sounds of the forest, the smell of plants and flowers, etc.) during the walk.
- The *second experimental group* took a FB in different forests of La Garrotxa area: Les Olletes (geographical coordinates: N 42° 9.886; E 2° 31.831) Serra d'Heures (geographical coordinates: N 41° 53.968; E 2° 31.683) and Can Fornaca (geographical coordinates: N 41° 48.200; E 2° 38.979). This group of participants was recruited in collaboration with Sèlvans Association (https://selvans.ong/). Sèlvans Association is a non-profit organisation that was born in 2007 with the main aim of conserving and adding value to the Catalan forests. It organises activities related to environmental education and nature conservation, such as FB. This group also did mindful walking in silence (without stops) being aware and opening their senses to facilitate nature connectedness (by listening the sounds of the forest, the smell of plants and flowers, etc.) around 2 h through the mentioned forests of La Garrotxa. It is located at the eastern end of the Catalan Pre-Pyrenees and is known as the volcanic area of Catalonia. Its geographical location makes it attractive for its proximity to the sea and to the mountainous regions of the country. This whole area is part of the Natura 2000 Network, an European initiative to protect the most unique, diverse, rare, well conserved, representative or vulnerable natural spaces. The climate of La Garrotxa is medium mountain Mediterranean. Rainfall is abundant throughout the year, and the winter is the driest season. The frequent showers keep summers cool, while the influence of the Pyrenees makes the winters very cold. The vegetation reflects the climate. While in the Alta Garrotxa and the east of the county there is an area of typically Mediterranean vegetation, the rest of the county is covered with sub-mediterranean vegetation that becomes Atlantic in the most humid parts. Holm oaks are typical on the sunny hillsides of the eastern sector, with relatively dry and hot summers, the oak tree predominates in other parts, and the beech is found in the most humid sectors. On the Olot plain, the most characteristic natural species is the common oak, although nowadays its presence is limited to small woods found around the city.
- *Control group* It included two hiking groups that did not take a FB but practiced a group-guided activity such as hiking. Hiking is a walk, usually on trails or footpaths in the countryside. The activities were organised in collaboration with the Network of Natural Parks of Catalonia and the School of Nature La Muntada (https://www.lamuntada.cat/ca), located in the Vall d’Horta of Sant LLorenç Savall (geographical coordinates: N 41º 40.638; E 2º 1.864). One grup hiked around 8 km. from la Vall d’Horta to a small-mountain called els Emprius-la Roca Foradada with an unevenness/inclination of 250 m (https://parcs.diba.cat/web/agenda/-/sant-lloren%C3%A7-obac-els-emprius-el-queixal-corcat-i-el-cau-dels-emboscats). A second control group hiked around 3 km. without inclination, along a plain and flat route in la Vall d’Horta https://parcs.diba.cat/web/agenda/-/sant-lloren%C3%A7-obac-passejada-teatralitzada-joan-oliver-al-marquet-de-les-roques-1. The two groups of activity that are reported as control condition were selected from among the cathalog of recreational and sports activities offered in the catalogue of activities of the institutions that have promoted the forest bathing experiences analysed in this study. For the selection of these activities, the researchers set the following inclusion criteria a priori: (1) Being a guided activity, (2) practiced in the same geographical spaces, (3) qualitatively different from forest bathing, (4) with the same duration (2 h) and (5) intensity. Of the two selected groups, second one met the 5 criteria and the first one 4 of them (all except low intensity). This gave rise to a statistical control of this possible confounding variable that is explained later.

A call for participation was made through the website of the corresponding organisers. Prior registration was requested to participate in all the activities. All participants consented to collaborate voluntarily and provided informed consent before participating in the study. The same pre-post surveys were administered immediately before and after all the activities online via Google Forms©, but also using paper–pencil questionnaires in the cases in which no internet-connection was available. The ethics committee of the Autonomous University of Barcelona (CEEAH-UAB) approved the study protocol (reference code UAB5339).

### Participants

Ninety-five people signed up for the events. A final sample of 86 volunteers participated in the study, including 57 participants in the FB session (Sant Llorenç *n* = 34; la Garrotxa *n* = 23), and 29 in the control group of hiking practice (with inclination *n* = 18; plain *n* = 11). The final sample analysed recorded an average age of 49.6 years (ranging from 19 to 78). Most of participants were residents in urban areas (75.6%), were women (69.8%) and had higher education (76,7%) (see Table 1).

**Table 1:** Descriptive statistics of the study participants

	Study sample (*n* = 86)*
Age *M* ± SD (Range)	49.02 ± 11.91 (19–78)
Gender, *n* women (%)	60 (69.8)
*Education, n (%)*	
Secondary	19 (22.4)
Tertiary	66 (77.6)
*Origin area, n (%)*	
Urban	65 (76.5)
Rural	20 (23.5)
*Location and Activity, n (%)*	
FB Sant Llorenç	34 (39.5)
FB Garrotxa FB	23 (26.7)
HI Sant Llorenç	18 (20.9)
HP Sant Llorenç	11 (12.8)

*FB* forest bathing, *HI* hiking with inclination, *HP* hiking plain

^*^Education and origin (*n* = 85); secondary (16 years), tertiary (around 25 years)

### Instruments

Both FB and hiking participants responded 4 standardised tests before and after the FB session. These questionnaires are widely used in the study of the psychological effects of FB and are cross-culturally validated for both the general and clinical populations (Hansen et al. 2017; Oh et al. 2017; Timko-Olson et al. 2020; Wen et al. 2019):

#### State-trait anxiety inventory

(STAI; Guillén-Riquelme and Buela-Casal (2011); Spielberger et al. 1982): This test measures the levels of state (situational) and trait (global personality) anxiety with a total of 20 items in each scale and format of Likert-type answers. In the present study, only state-anxiety was measured. High scores warn of altered states related to anxiety, low scores indicate emotional stability and absence of stress. In the original Spanish validation, STAI-state showed an internal consistency of Cronbach’s *α* = .90. In the study sample, STAI-state showed an internal consistency of Cronbach’s *α* = .88.

#### Positive affect and negative affect scale

(PANAS; Watson et al. 1988; López-Gómez et al. 2015): It includes two subscales of 10 items each that assess the experience of positive emotions related to psychological well-being and experiencing negative emotions related to psychological distress and mental health problems. In the original Spanish validation, positive affect subscale showed an optimal internal consistency of Cronbach’s *α* = .88, and negative affect subscale showed an internal consistency of Cronbach’s *α* =. 87. In the study sample, positive affect subscale showed an optimal internal consistency of Cronbach’s *α* = .91, and negative affect subscale showed an internal consistency of Cronbach’s *α* =. 910.

#### Profile of mood states

(POMS; McNair et al. 1971; Andrade et al. 2010). This test measures 6 moods from 30 items: Anger, fatigue, vigour, friendship, tension, and depressive mood; each item can be scored in a scale ranging from 0 to 4. In the original Spanish validation, the subscales showed an internal consistency of Cronbach’s ranging from *α* = .77 to Cronbach’s *α* = .92 for the six subscales. In the study sample, the subscales showed an internal consistency of Cronbach’s ranging from *α* = .92 to Cronbach’s *α* = .96 for the six subscales.

#### State mindfulness scale

(M-E; Tanay and Bernstein (2013)): It is composed of 21 items with a Likert-type response scale to indicate whether the sentences describe well their experiences in the last 15 minutes. It assesses two dimensions: (1) mindfulness state of mind (e.g. "I realized thoughts coming and going") and (2) mindfulness state of the body (e.g. "I realized physical sensations coming and going"). In the original Spanish validation, mind mindfulness subscale showed an optimal internal consistency of Cronbach’s *α* = .95, and body mindfulness subscale showed an internal consistency of Cronbach’s *α* = .90. General score of mindfulness also showed an optimal internal consistency of Cronbach’s *α* = *.95.* In the study sample, mind mindfulness subscale showed an optimal internal consistency of Cronbach’s *α* = .94, and body mindfulness subscale showed an internal consistency of Cronbach’s *α* = .90. General score of mindfulness also showed an optimal internal consistency of Cronbach’s *α* = *.96.*

### Data preparation and statistical analyses

Statistical analyses were conducted using the IBM-SPSS 26.0 statistical package. No participants had to be discarded due to significant data loss. The omission of answers in some of the participants in the little group of paper-and-pencil data gathering procedure (less than 0.5%) explains the slight differences in the degrees of freedom indicated in the hypothesis tests. Prior to the analyses, an evaluation of the quality of the raw data obtained was carried out in two steps. In the first step, a principal components analysis (PCA) was carried out with a VARIMAX rotation, an extraction of the theoretical factors reported in the respective psychometric validations, and a selection of factor loadings greater than 0.40. In all cases, a satisfactory factorial structure (matching the theoretical one) was obtained. In the second step, a reliability analysis was performed using Cronbach's alpha internal consistency index for all scales and subscales. As reported in the instruments section, internal consistency indices obtained were in the range of Cronbach’ *α* = 0.77 and Cronbach’ *α* = 0.96, thus very satisfactory and comparable to those of the original validations of the respective instruments. The empirical structure of all the measurement scales was identical to the theoretical, except for the friendship subscale of the POMS, from which one of the items was eliminated due to a significant reduction in reliability; this subscale values have been rescaled to the original range [0–20] for a better interpretation of the results.

Once the data quality tests had been carried out, the scales’ scores were calculated, as an identification of a possible confounding variable, the homogeneity of the two control groups was checked regarding the level of intensity of the hiking carried out. An analysis with a general linear model of mixed design revealed the absence of differences between both groups in the pre-post evolution for state-anxiety, positive affect, negative affect, the six POMS subscales and body mindfulness. Significant differences were found between both groups in mindfulness of mind (Wilk' *λ*_(1,27)_ = 4.55; *p* = 0.04; *η*^2^ = 0.14) and in the general mindfulness scale (Wilk' *λ*_(1,27)_ = 4.73; *p* = 0.04; *η*^2^ = 0.15), so that the activity intensity variable was generated, which was included as a covariate in the following statistical analyses of these two variables.

## Results

### Cognitive and affective effects of the practice of forest baths vs. hiking

**Fig. 1:**
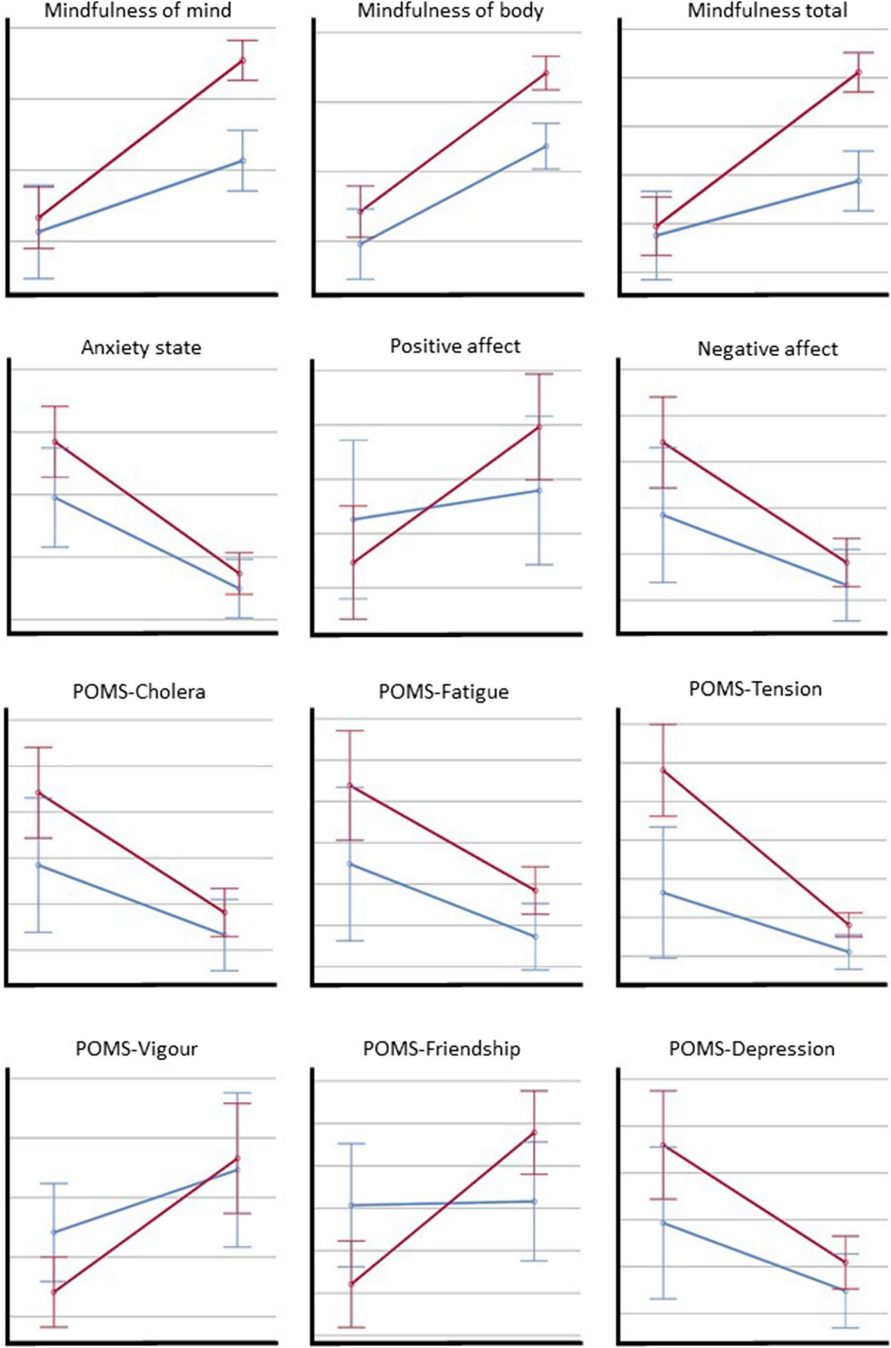
Marginal estimated means and CI 95% for pre-post assessment of cognitive and affective variables. Comparing between experimental group (red line) and control group (blue line). Marginal estimated means for mindfulness of mind and mindfulness total were obtained including intensity as a covariate to control baseline differences

Socio-demographics and descriptives of the study participants can be seen in Table 1. A general linear model of analysis of variance (mixed design) was carried out with a within-subject factor (pre- vs. post-phase) and a between-groups factor (experimental vs. control group) on all the variables evaluated (see Table 2 and Fig. 1). A statistically significant pre-post change was observed for all the variables in the sample as a whole: an increase in mindfulness of mind, body and global, positive affect vigou**r** and friendship, as well as a reduction in state-anxiety, negative affect, anger, fatigue, tension and depression were observed.

**Table 2:** General linear models for cognitive and affective effects of experimental intervention group (guided forest bathing) versus control group (guided hiking)

				Group			Phase			Interaction group*Phase		
		FB (*n* = 57)^*^ *M* ± SD	Hiking (*n* = 29)^**^ *M* ± SD	*F*	*p*	*ɳ*_p_^2^	*F*	*p*	*ɳ*_p_^2^	*F*	*p*	*ɳ*_p_^2^
*STAI-S*												
State-anxiety	Pre	19.23 ± 12.02	14.76 ± 7.6	2.43	.123	.03	107.71	< .001	.56	3.62	.06	.04
	Post	8.68 ± 7.09	7.48 ± 4.45									
*PANAS*												
Negative affect	Pre	18.84 ± 8.15	15.69 ± 5.73	3.06	.084	.04	27.53	< .001	.25	1.91	.171	.02
	Post	13.63 ± 4.53	12.65 ± 2.24									
Positive affect	Pre	32.93 ± 8.62	34.52 ± 6.26	.06	.802	.001	11.68	.001	.12	4.90	.03	.06
	Post	37.93 ± 8.34	35.59 ± 5.03									
*POMS*												
Anger	Pre	2.33 ± 4.24	.35 ± 1.05	6.83	.011	.09	9.11	.003	.10	4.51	.04	.05
	Post	.35 ± 1.26	.00 ± 0									
Fatigue	Pre	4.39 ± 5.48	2.48 ± 3.99	4.42	.039	.05	17.48	< .001	.17	.58	.45	.01
	Post	1.8 ± 2.51	.72 ± 1.28									
Vigour	Pre	10.82 ± 4.65	12.83 ± 4.05	.49	.49	.01	23.70	< .001	.22	3.12	.082	.04
	Post	15.32 ± 7.74	14.93 ± 5.30									
Friendship	Pre	14.21 ± 4.31	16.07 ± 2.74	.02	.877	0	18.08	< .001	.18	16.36	< .001	.17
	Post	17.79 ± 3.40	16.16 ± 4.34									
Tension	Pre	4.81 ± 5.21	1.64 ± 2.50	11.02	.001	.12	31.86	< .001	.28	6.31	.014	.07
	Post	.81 ± 1.42	.11 ± .31									
Depression	Pre	3.60 ± 4.97	1.93 ± 2.89	2.95	.09	.03	21.51	< .001	.20	1.55	.217	.02
	Post	1.09 ± 2.14	.48 ± .91									
*SMS*												
Mindfulness of the Mind	Pre	44.26 ± 14.42	39.59 ± 11.96	8.82	.004	.10	26.92	< .001	.25	5.13	.03	.06
	Post	64.19 ± 9.07	53.66 ± 8.74									
Mindfulness of the Body	Pre	16.56 ± 5.90	15.48 ± 5.78	18.60	< .001	.19	56.74	< .001	.41	15.27	< .001	.16
	Post	25.26 ± 4.67	18.24 ± 4.32									
Mindfulness General	Pre	60.82 ± 19.67	55.07 ± 17.23	9.72	.003	.11	25.40	< .001	.24	7.84	.006	.09
	Post	89.44 ± 13.21	71.90 ± 12.29									

*FB* forest bathing, *PANAS* positive affect and negative affect scale, *POMS* profile of mood states, *SMS* state mindfulness scale, *STAI-S* state-trait anxiety inventory (state subscale)

^*^Mindfulness of the mind, mindfulness of the body and mindfulness general (*n* = 54); ** friendship and tension (*n* = 28)

All statistical significances were equal or less than *p* = 0.003, and effect sizes ranged from *η*_*p*_^2^ = . 10 (POMS-anger) to *η*_*p*_^2^ = 0.56 (STAI-state-anxiety). Group x phase interactions revealed a greater pre-post increase in the experimental condition for mindfulness of mind (Wilks' *λ*_(1,80)_ = 5.13; *p* = 0.03; *η*_*p*_^2^ = 0.06, controlling for intensity), for mindfulness of body (Wilks' *λ*_(1,81)_ = 15.27; *p* < 0.001; *η*_*p*_^2^ = 0.16), total mindfulness (Wilks' *λ*_(1,80)_ = 7.84; *p* = 0.006; *η*_*p*_^2^ = 0.09, controlling for intensity), positive affect (Wilks' *λ*_(1,84)_ = 4.90; *p* = 0.03; *η*_*p*_^2^ = 0.06), friendship (Wilks' *λ*_(1,83)_ = 16.36; *p* < 0.001; *η*_*p*_^2^ = 0.17), as well as a greater reduction in state-anxiety (Wilks' *λ*_(1.84)_ = 3.62; *p* = 0.06; *η*_*p*_^2^ = 0.04), anger (Wilks' *λ*_(1.81)_ = 4.51; *p* = 0.04; *η*_*p*_^2^ = . 05) and tension (Wilks' *λ*_(1.83)_ = 6.31; *p* = 0.014; *η*_*p*_^2^ = 0.07).

### Differences in cognitive and affective effects depending on the FB’s sub-biome

Given that the groups that practised guided forest bathing were carried out in two different biomes, a specific sub-analysis of the experimental group was carried out to identify the possible differences in the effects observed in both (see Table 3). Given that the very nature of the study prevented the random assignment of the participants, a prior comparison of the baseline values was made, in case it was necessary to declare the confounding variables and carry out a statistical control through analysis of covariance. Significant differences were found in the baseline values of the Sant Llorenç (pre-coastal Mediterranean) and La Garrotxa (mountain Mediterrean) groups in state-anxiety (*F* = 7.33; *p* = 0.009), anger (*F* = 5.58; *p* = 0.022), tension (*F* = 9.96; *p* = 0.003), depression (*F* = 10.31; *p* = 0.002) and negative affect (*F* = 4.69; *p* = 0.035), with consistently higher values in baseline values in these variables in the Sant Llorenç group. The variance analysis showed a better evolution after the nature activities of this group for all these variables. However, once the pre value was introduced as a covariate, the differences in the pre-post evolution between both groups disappeared for all the variables, except in state-anxiety, (*F*_(1,54)_ = 15.02; *p* < 0.001; *η*_*p*_^2^ = 0.21) and tension (*F*_(1,54)_ = 9.56; *p* = 0.003; *η*_*p*_^2^ = 0.15) which showed higher effects for the group that carried out the FB in Sant Llorenç*.*

**Table 3:** General linear models for cognitive and affective effects comparing experimental groups (Garrotxa vs. Sant Llorenç)

				Group			Phase			Interaction Group*Phase		
		FB Sant Llorenç (*n* = 34) *M* ± SD	FB Garrotxa (*n* = 23) *M* ± SD	*F*	*P*	*ɳ*_p_^2^	*F*	*p*	*ɳ*_p_^2^	*F*	*p*	*ɳ*_p_^2^
*STAI-S*												
State-anxiety	Pre	22.58 ± 12.52	14.26 ± 9.43	3.82	.056	.06	108.34	< .001	.66	15.02	< .001	.21
	Post	9.08 ± 7.22	8.08 ± 7.01									
*PANAS*												
Negative affect	Pre	20.70 ± 8.66	16.08 ± 6.57	5.66	.021	.09	28.43	< .001	.34	1.54	.220	.03
	Post	14.56 ± 4.61	12.26 ± 4.11									
Positive affect	Pre	32.97 ± 7.78	32.86 ± 9.91	.24	.628	.004	18.04	< .001	.25	.59	.44	.01
	Post	38.67 ± 6.47	36.82 ± 10.59									
*POMS*												
Anger	Pre	3.38 ± 4.76	.78 ± 2.75	5.21	.026	.87	10.89	.002	.16	5.00	.03	.08
	Post	.44 ± 1.39	.21 ± 1.04									
Fatigue	Pre	5.44 ± 5.96	2.82 ± 4.32	3.75	.058	.06	12.46	.001	.18	1.61	.21	.03
	Post	2.20 ± 2.72	1.30 ± 2.07									
Vigour	Pre	10.26 ± 4.26	11.65 ± 4.90	.004	.95	.00	22.68	< .001	.29	.004	.94	.00
	Post	14.70 ± 4.69	16.21 ± 10.86									
Friendship	Pre	14.55 ± 4.02	15.47 ± 10.30	.004	.94	.00	9.36	.003	.14	.925	.34	.02
	Post	18.05 ± 2.22	17.30 ± 4.58									
Tension	Pre	6.47 ± 5.47	2.34 ± 3.67	8.99	.004	.14	35.18	< .001	.39	9.56	.003	.15
	Post	.94 ± 1.27	.60 ± 1.61									
Depression	Pre	5.20 ± 5.60	1.21 ± 2.39	11.55	.001	.17	16.77	< .001	.23	4.58	.037	.07
	Post	1.73 ± 3.12	.13 ± .34									
*SMS*												
Mindfulness of the mind	Pre	45.38 ± 15.59	42.35 ± 12.32	.637	.428	.12	63.58	< .001	.55	.22	.63	.00
	Post	64.41 ± 9.97	63.80 ± 7.51									
Mindfulness of the body	Pre	17.29 ± 5.87	15.30 ± 5.88	1.89	.175	.03	81.24	< .001	.61	.20	.66	.00
	Post	25.67 ± 4.57	24.55 ± 4.86									
Mindfulness general	Pre	62.67 ± 20.82	57.65 ± 17.58	1.08	.304	.02	71.31	< .001	.58	.23	.63	.00
	Post	90.08 ± 14.06	88.35 ± 11.89									

*FB* forest bathing, *PANAS* positive affect and negative affect scale, *POMS* profile of mood states, *SMS* state mindfulness scale, *STAI-S* state-trait anxiety inventory (state subscale)

### Profiles of people practising forest bathing vs hiking

A univariate analysis of variance was carried out to compare the baseline values of the variables evaluated between the experimental group. Differences were found in the baseline values of POMS-cholera (*M*_hiking_ = 0.34 vs. *M*_forestbath_ = 2.33; *F*_(1,84)_ = 6.17; *p* = 0.01), POMS-vigour (*M*_hiking_ = 12.34 vs. *M*_forestbath_ = 10.82; *F*_(1,84)_ = 3.88; *p* = 0.05), POMS-friendship (*M*_hiking_ = 16.07 vs. *M*_forestbath_ = 14.21; *F*_(1,84)_ = 4.35; *p* = 0.04) and POMS-tension (*M*_hiking_ = 1.64 vs. *M*_forestbath_ = 4.80; *F*_(1,84)_ = 9.24; *p* = 0.003), so they were included as covariates of the respective hypothesis tests as statistical control. No statistically significant differences were found in the rest of the variables evaluated. With regard to the sociodemographic variables, it was found that there are differences in gender (women 77% of the forest bathers vs. 55% of the hiking practitioners; *χ*^2^ = 4.41; *p* = 0.03) and in the level of studies (university students 84% of the forest bathing practitioners vs. 66% of the hiking practitioners; *χ*^2^ = 3.73; *p* = 0.05). No differences were found in age, origin (urban vs. rural) or in previous pathologies.

## Discussion and conclusions

The present study aimed to explore whether FB in Mediterranean forests would significantly increase psychological well-being in a sample of healthy adults and to contrast its effects with a control-hiking group. It was also aimed to test if there was a differential effect among the FB performed in two different Mediterranean sub-biomes of the Catalan territory during the COVID-19 pandemic. The main results of this study are in line with those reported robustly in Asian biomes (Hansen et al. 2017; Li 2018; Timko-Olson et al. 2020; Wen et al. 2019) and show how FB in Mediterranean regions also enhance mood states by decreasing anxiety, negative affect, tension, colera, fatigue and depression indicators, and by increasing mindfulness, positive affect, vigour and friendship outcomes, a result which is also in line with a pilot study in Mediterranean biomes (Muro et al. 2022). An improvement was obtained in all the affective and cognitive processes evaluated within subjects participating in the FB, with effect sizes ranging from moderate to very large, the latter being the predominant ones. Therefore, results are consistent with hypothesis 1 and add more evidence to universal benefits of FB for mental health in different biomes of the world and thus for the therapeutic effects of this nature activity (Kotera et al. 2020). It is worth noting that significant changes have been observed in all the psychological indicators measured in the present study, with large effect sizes after one single FB session. The highest impact has been shown in anxiety and tension decreases, as well as increases in vigour, a result that is also in line with physiological studies reporting lower salivary cortisol levels in FB participants, a solid marker of relaxation and stress reduction (Antonelli et al. 2019; Bach et al. 2020a, b; Chen et al. 2018; Park et al. 2010). It is also remarkable that although participants were walking around 4 km., fatigue decreased and vigour increased, suggesting that walking in plain territory is a physical activity with revitalising properties that also facilitates physical well-being and vitality. Furthermore, large increases in mindfulness indicators have been found (both for mindfulness of the body and the mind) after the FB, pointing out how mindfulness states and its sensory and cognitive process are highly enhanced through this silent walk by the forest. Thus, FB could be regarded as an outdoor-mindfulness practice that could be included in psychological interventions (Sadowski et al. 2020; Timko-Olson et al. 2020; van Gordon et al. 2018). It is also remarkable the changes observed in friendship feelings, highlighting how guided forest activities increase social connectedness, even if they are performed in silence, without allowing participants talk while they are walking through the forest. Thus, results observed in this study confirm that Mediterranean FB is not only a touristic and nature-guided activity that allows visitors discover the landscape under a sustainable approach (Farkic et al. 2021), but it can also be regarded as a powerful nature activity that increases positive mental health and well-being indicators at the short therm. Accordingly, it is concluded that Mediterranean FB might also be a cost-effective intervention for promoting emotional well-being and sustainable tourism (Farkic et al. 2021; Ohe et al. 2017). FB promotion could contribute to facilitate actions and agreements of the European Charter for Sustainable Tourism (ECST) the protected areas of Mediterranean-Catalan regions (Canals 2014; Europarc Federation 2010). It is worth noting that both basal levels of stress indicators and effect sizes were higher in the Sant Llorenç group of FB, a result that was somehow expected, since Sant Llorenç forests are closer to Barcelona metropolitan area, and its participants were mostly living in this area, while participants of La Garrotxa were mostly living in rural areas. This result is also consistent with previous studies reporting how urban populations show higher levels of stress and decreased well-being, and thus, FB might have a more powerful impact in urban populations (Antonelli et al. 2019; Morita et al. 2007; Oh et al. 2017; Roviello and Roviello 2020; Wen et al. 2019).

Regarding the second hypothesis, the comparison with the hiking group indicates that this guided nature activity also produces positive changes for psychological well-being (Mitten et al. 2018), but the effect sizes are lower than in FB, probably because the latter is rather designed with well-being goals and might appeal specific populations who are looking for more therapeutic nature activities. Accordingly, it is suggested that the additive effect of silence and the easiness of the plain route might explain the differential and more powerful effect of the FB activity, as well as the differential participants’ profile: FB practitioners, compared to hiking ones are mainly women living in urban areas, with higher basal values of tension/anxiety and anger, and lower values of friendship and vigour. In this way, hypothesis 2 has been partially verified since higher effect sizes have been reported after the FB. Considering all constructs of the spectrum, the negative valence (anxiety, tension, depression, fatigue, anger) as a whole and the distances in the benefit obtained between both activities tend to be smaller in the hiking group. This result might highlight the fact that FB attracts individuals (women) living in urban areas that show less psychological well-being, and thus can benefit the most of nature immersion through guided activities. Therefore, in line with previous studies (Buckley and Westaway; 2020), our results show that nature activities could be cost-effective solutions to accelerate women’s psychological recovery after the COVID-19. It is worth noting that women’s mental health has been more hit by the pandemic than men’s (Muro et al. 2021; Sanabria-Mazo et al. 2021) and the virus has also impacted less those populations living in forested areas (Roviello and Roviello, 2020). Therefore, FB could be considered a gender-focused approach targeted at vulnerable groups for decreasing mental health after the pandemic, such as women and youngest populations.

Finally, regarding the second exploratory objective of this study, no differences were found in the practice of forest bathing in the different allocations of this study, characterised by two different sub-biomes of the Mediterranean-Catalan area. Although in a first analysis some differences were found suggesting a higher impact of the Sant Llorenç FB, a control of confounding variables neutralised these differences. It should be noted that the higher impact observed in FB of Sant Llorenç could be better explained, as commented above, by the participants profile, rather than forests’ sub-biomes: participants of Sant Llorenç were living closer to metropolitan areas of Barcelona surroundings and showed worse baseline levels in the different mental health indicators, so they probably benefited from the restorative effects of FB to a greater extent.

### Limitations and future directions

Nevertheless, several limitations of the present study should be acknowledged. Firstly, it is necessary to highlight the fact that this study has only evidenced short-term psychological effects of one single FB, a limitation that is widely repeated on FB research. Thus, larger FB programmes should be tested to explore long-term impacts on mental health outcomes, including follow-up assessments. More research should also be carried not just using larger FB programmes, but also analysing clinical samples at higher risk for stress-related or anxiety disorders.

An important strength of this study is that, on the intra-subject analysis carried out on the participants in the forest baths, it has been possible to incorporate the parallel evaluation of a hiking-control group, since hiking is considered a nature activity with numerous health benefits (Mitten et al. 2018). This control group fully met 4 of 5 criteria set by the researchers to reduce bias in the interpretation of those results that imply a comparison between both conditions: being a guided activity, practiced in the same geographical spaces, qualitatively different from forest bathing and with the same duration. It was not possible to fully meet the fifth criterion, which consisted in the physical load being of a low intensity, comparable to that exerted in FB. That was because one of the hiking groups did a higher intensity activity. However, this fact was recorded and conveniently neutralised through the physical load intensity variable as covariates of the developed statistical models. Furthermore, despite having a control group, the fact that it was implemented on the frame of nature experiences promoted by various public and private institutions (with different recruitment and advertising processes) and being essentially a community service, it was not possible to carry out any type of randomisation. Nevertheless, this weakness was an opportunity to explore the possible bias in relation to the characteristics of the population that chose FB instead of other options for practicing sports in nature. Finally, we should also remark that all measures included in this pilot study assessed cognitive and emotional states (i.e. right now) that are highly variable. Thus, further works should assess programmes with various sessions of FB and include objective indicators of anxiety or stress such as cortisol (Bach et al. 2021), variables with larger evaluation timeframes (e.g. last week), which are normally preferred as main outcomes in psychotherapy research to assess long-term benefits. Thus, it would be better to reach more robust and consistent conclusions on the benefits of FB for overall psychological functioning and mental health (Kotera et al. 2020). In this regard, future studies comparing larger FB programmes and nature activities with “standard” psychological interventions are particularly welcome.

## Conclusions

The present study adds more empirical evidence to both FB practice and thus to nature-medicine health model (Bratman et al. 2019; Hansen et al. 2017; Twohig-Bennett and Jones 2018). It aligns with previous studies in other forested areas of the world showing that FB is a powerful outdoor activity that facilitates psychological well-being, and followed by hiking, can be considered as sustainable touristic activities that could add an a relevant additive value to Mediterranean-European forested and protected areas. In any case, this study encourages further research and implementations of FB in other European forested areas as a cost-effective public-health strategy to cope with the increasing mental health problems after the pandemic among urban populations, especially among women (Antonelli et al. 2019; Dubey et al. 2020; Muro et al. 2021; Sanabria-Mazo et al. 2021; Wang et al. 2020). Finally, it is suggested that the inclusion of FB in European forests could also be a cost-effective action that could contribute to reach the goals of the European Charter for Sustainable Tourism in Protected Areas (Canals 2014; Europarc Federation 2010; Wanner et al. 2020) in terms of promoting sustainable activities that facilitate well-being and pro-environmental attitudes through nature connectedness.
